# Does Vancomycin Resistance Increase Mortality? Clinical Outcomes and Predictive Factors for Mortality in Patients with *Enterococcus faecium* Infections

**DOI:** 10.3390/antibiotics10020105

**Published:** 2021-01-22

**Authors:** Jatapat Hemapanpairoa, Dhitiwat Changpradub, Sudaluck Thunyaharn, Wichai Santimaleeworagun

**Affiliations:** 1 Department of Pharmacy Practice and Pharmaceutical Care, Faculty of Pharmaceutical Sciences, Burapha University, Chonburi 20131, Thailand; aumloveka@gmail.com; 2 Pharmaceutical Initiative for Resistant Bacteria and Infectious Disease Working Group [PIRBIG], Nakorn Pathom 73000, Thailand; 3 Division of Infectious Disease, Department of Medicine, Phramongkutklao Hospital, Bangkok 10400, Thailand; dhitiwat@yahoo.com; 4 Faculty of Medical Technology, Nakhonratchasima College, Nakhon Ratchasima 30000, Thailand; tanmicro@gmail.com; 5 Department of Pharmacy, Faculty of Pharmacy, Silapakorn University, Nakorn Pathom 73000, Thailand

**Keywords:** *Enterococci*, survival, risk factor, VRE, glycopeptide

## Abstract

The prevalence of enterococcal infection, especially *E. faecium,* is increasing, and the issue of the impact of vancomycin resistance on clinical outcomes is controversial. This study aimed to investigate the clinical outcomes of infection caused by *E. faecium* and determine the risk factors associated with mortality. This retrospective study was performed at the Phramongkutklao Hospital during the period from 2014 to 2018. One hundred and forty-five patients with *E. faecium* infections were enrolled. The 30-day and 90-day mortality rates of patients infected with vancomycin resistant (VR)-*E. faecium* vs. vancomycin susceptible (VS)-*E. faecium* were 57.7% vs. 38.7% and 69.2% vs. 47.1%, respectively. The median length of hospitalization was significantly longer in patients with VR-*E. faecium* infection. In logistic regression analysis, VR-*E. faecium*, Sequential Organ Failure Assessment (SOFA) scores, and bone and joint infections were significant risk factors associated with both 30-day and 90-day mortality. Moreover, Cox proportional hazards model showed that VR-*E. faecium* infection (HR 1.91; 95%CI 1.09–3.37)*,* SOFA scores of 6–9 points (HR 2.69; 95%CI 1.15–6.29), SOFA scores ≥ 10 points (HR 3.71; 95%CI 1.70–8.13), and bone and joint infections (HR 0.08; 95%CI 0.01–0.62) were significant risk factors for mortality. In conclusion, the present study confirmed the impact of VR-*E. faecium* infection on mortality and hospitalization duration. Thus, the appropriate antibiotic regimen for VR-*E. faecium* infection, especially for severely ill patients, is an effective strategy for improving treatment outcomes.

## 1. Introduction

*Enterococcus*, a gram-positive cocci occurring in chains, is a major pathogen in community and nosocomial infections. The infections caused by the *Enterococci* spp. includes the genitourinary tract, intra-abdomen, bloodstreams, infective endocarditis, skin/soft tissue, rarely bone and joint, and central nervous system. The species that are clinically important and the cause of most infections are *E. faecalis* and *E. faecium* [1]. However, *E. faecalis* is a common cause of community and in-hospital infection, while most *E. faecium* causes nosocomial infection. Additionally, *E. faecium* is generally resistant to penicillin (with ampicillin being a drug of choice for treatment of enterococcal infection) by the production of beta-lactamase and point mutation in penicillin-binding proteins (PBPs); it is occasionally reported resistant to vancomycin (also called vancomycin resistant *Enterococci*; VRE), by modifying pentapeptide precursors [1,2].

VRE isolates have been widely reported [3]. As per data from the National Healthcare Safety Network from 2009 to 2010, around 1/3 of all enterococcal associated nosocomial infections are VRE. It is noteworthy that VRE is the second most common cause of nosocomial infections in the United States of America [4,5]. As per the National Antimicrobial Resistance Surveillance, Thailand data, an increased prevalence of VRE was reported, from 0.4% in 2012 to 6.4% in 2019, especially that of *E. faecium* that is resistant to vancomycin, which was 7.2% in 2019 [6].

Importantly, *Enterococci* can cause serious infection in debilitated patients. Patients can acquire enterococcal infections from colonization and the hospital environment. Patients infected with enterococci had a mortality rate of 32%–66.7% [7,8,9,10,11,12,13,14] depending on their prognostic factors, such as underlying diseases, Charlson Comorbidity Index (CCI), illness severity (Acute Physiology and Chronic Health Evaluation; APACHE) II, Pitt bacteremia, Organ System Failure Index; OSFI), admission to the intensive care unit (ICU), shock, active drug against VRE, and particularly infection caused by vancomycin-resistant strains [12,14,15,16,17].

However, several previous studies have shown that VRE infection is not associated with increased hospital deaths [18,19,20,21]. Whether the mortality among patients with VRE infection is higher than that among those with VSE remains a controversial issue. Additionally, certain studies described above gathered patient outcomes of *E. faecium* mixed with *E. faecalis*, and occasionally *E. gallinarum* [7,8,9,16,22]. Infection due to VR-*E. faecium* was associated with greater than 2 fold-higher risk of mortality as compared with infections caused by VR-*E. faecalis* [16].

Owing to the growing prevalence of enterococcal infection, especially *E. faecium,* the controversial impact of vancomycin resistance on clinical outcomes, and the available data pertaining non-specifically to infections due to *E. faecium*, this study aimed to investigate the clinical outcomes of infection due to *E. faecium* and determine the risk factors associated with mortality, including the risk attributed to vancomycin resistance.

## 2. Results

During the period from 2014 to 2018, there were 145 patients with *E. faecium* infection. Of these, 80 (55.2%) were men, and the median patient age was 72 years (IQR 22 years). The median SOFA score was 5 points (IQR 7 points). Sixty (41.4%) patients were admitted to the intensive care unit, and 71 (49.0%) required mechanical ventilation. The most common comorbidity among patients with *E. faecium* infections was solid tumors (38.6%), followed by cardiovascular diseases (30.3%) and cerebrovascular diseases (17.9%).

### 2.1. Clinical Outcomes in Patients with VR-E. faecium and VS-E. faecium Infection

The prevalence of VS-*E. faecium* and VR-*E. faecium* infection was 119 (82.1%) and 26 (17.9%), respectively. The baseline characteristics of the patients infected with VS-*E. faecium* and VR-*E. faecium* infection are shown in Table 1. Male sex, end stage kidney disease, bone/joint infection, and urinary tract infection were significantly different between patients infected with VS-*E. faecium* and VR-*E. faecium* infection.

**Figure 1:**
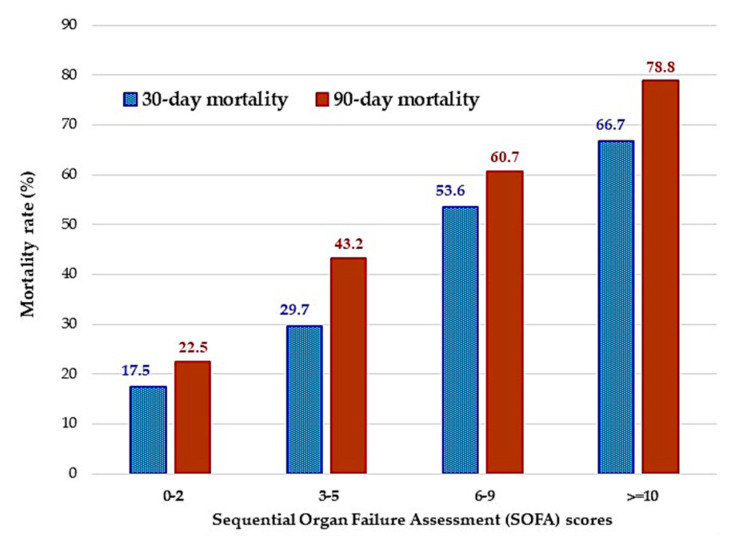
30–day and 90–day mortality rates categorized by Sequential Organ Failure Assessment (SOFA) scores (0–2, 3–5, 6–9, and ≥10 points).

The 30-day and 90-day mortality rates in patients infected with VR-*E. faecium* vs. VS-*E. faecium* were 57.7% vs. 38.7% and 69.2% vs. 47.1%, respectively (Table 1). The 30-day and 90-day mortality rates categorized by SOFA scores (0–2, 3–5, 6–9, and ≥10 points) are shown in Figure 1. The in-hospital mortality rates for these patients were 73.1% and 49.6%, respectively. The in-hospital mortality (*p* = 0.03) and 90-day mortality rate (*p* = 0.04) was significantly different between patients with VS-*E. faecium* and VR-*E. faecium* infection. The median length of hospitalization was significantly longer in patients with VR-*E. faecium* infection than in those with VS-*E. faecium* (69 days vs. 36 days, *p* = 0.001).

### 2.2. Risk Factors for 30-Day and 90-Day Mortality

As per the univariate analysis for 30-day and 90-day mortality, those who died were aged ≥ 70 years, had higher SOFA scores, had VRE infection, or had bacteremia. However, the prevalence of bone and joint infection was higher in the surviving patients. In logistic regression analysis, VR-*E. faecium* (aOR 3.64; 95%CI 1.20–11.07)*,* SOFA scores of 6–9 points (aOR 4.61; 95%CI 1.43–14.87), and SOFA scores ≥ 10 points (aOR 6.94; 95%CI 2.23–21.59), and bone and joint infections (aOR 0.09; 95%CI 0.01–0.91) were significant risk factors associated with 30-day mortality (Table 2). However, age ≥ 70 years (aOR 3.56; 95%CI 1.50–8.48), VR–*E. faecium* (aOR 7.35; 95%CI 1.79–30.21), SOFA scores of 6–9 points (aOR 4.40; 95%CI 1.34–14.48), SOFA scores ≥ 10 points (aOR 9.78; 95%CI 2.93–32.70), and bone and joint infections (aOR 0.034; 95%CI 0.02–0.49) were significant risk factors associated with 90-day mortality (Table 3).

### 2.3. Cox Proportional Hazard Regression Analysis of 90-Day Survival

**Figure 2:**
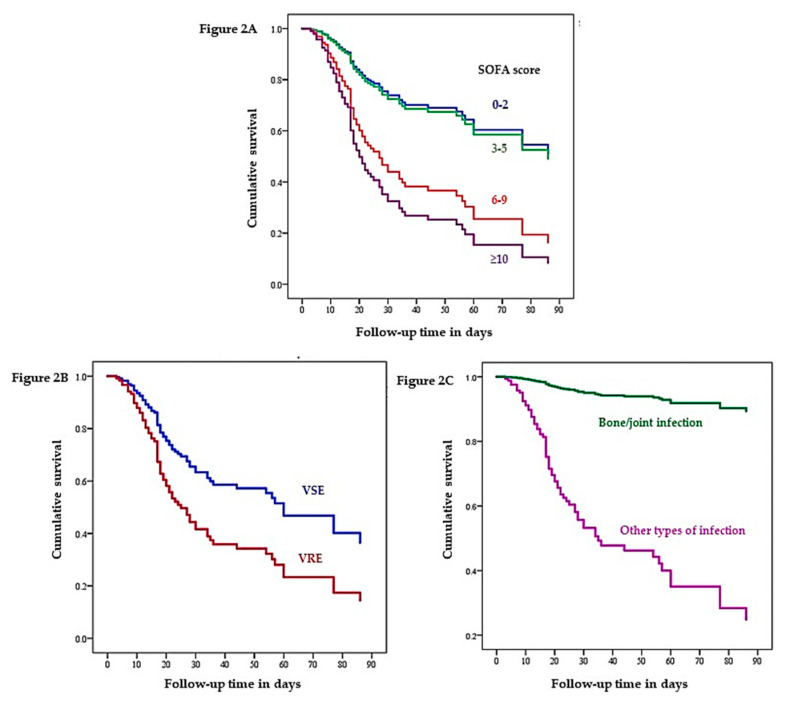
Cox proportional hazards cumulative 90-day survival curves with respect to different SOFA score groups (**A**), vancomycin susceptibility pattern (**B**), and bone/joint infection vs. other type of infections (**C**) after sepsis adjusted for other significant variables (age ≥ 70 years, vancomycin resistance, bone/joint infection, or bloodstream infection). Abbreviations: SOFA, sequential organ failure assessment; VRE, vancomycin resistant *Enterococci*; VSE, vancomycin susceptible *Enterococci*.

The factors related to 90-day survival that were significant on univariate analysis were further evaluated using a Cox proportional hazards model, and the hazard ratios were calculated. On multivariate analysis, VR-*E. faecium* (HR 1.91; 95%CI 1.09–3.37)*,* SOFA scores of 6–9 points (HR 2.69; 95%CI 1.15–6.29), SOFA scores ≥ 10 points (HR 3.71; 95%CI 1.70–8.13), and bone and joint infections (HR 0.08; 95%CI 0.01–0.62) were significant risk factors for mortality (Table 4). The Cox proportional hazards cumulative 90-day survival curves with respect to different SOFA scores, vancomycin susceptibility pattern, and bone/joint infection vs. other organ infections are shown in Figure 2A–C, respectively.

## 3. Discussion

Previously, several reports showed that patients with VRE versus those with VSE infection did not have a significantly greater risk of mortality [19,20,21]. In contrast, our study reported 30-day and 90-day mortality rates for VR-*E. faecium* infection patients of 57.7% and 69.2%, respectively; these values were obviously higher than those in patients with VS-*E. faecium* infection cases (38.7% and 47.1%). These results were similar to previous studies indicating the clinical impact of VRE on patient outcomes and hospitalization duration [14,23].

Moreover, the median length of hospitalization was significantly longer in patients with VR-*E. faecium* infection than in those with VS-*E. faecium* (69 days vs. 36 days). Prematunge et al. performed a meta-analysis of VRE and VSE bacteremia outcomes among hospital patients in the era of effective VRE therapy [24]. Among all the studies that were reviewed, the length of stay (LOS) was significantly longer in the VRE group than in the VSE group (mean difference, 5.01 days; 95% CI, 0.58–9.44]) [24]. Similarly, we found an obvious increase in the duration of hospitalization among VR-*E. faecium* infection patients. Considering only the patients infected with VR-E. *faecium*, we also confirmed that VRE infection remained associated with an increased LOS. Therefore, the present study also conformed the influence of VR-*E. faecium* infection on patients. However, due to variable findings across previous studies, the role for vancomycin resistance in clinical significance has to be further evaluated.

As per multivariate and cox-regression analyses, the mortality rate was higher in patients with severe illness based on the SOFA scores and VR-*E. faecium* infection. A higher severity index score was a direct independent risk factor for mortality. Similar to previous reports [8,18,20], we also found that the SOFA score increased the risk of poor outcome. Moreover, as for the type of infection, bone and joint infection was classified as decreasing the risk of death. Terpenning et al. [25] indicated that the most common sites for *Enterococci* isolation were the urinary tract, bone and soft tissue; however, the overall mortality rate in patients with bacteremia was as high as 71.4%.

To our knowledge, no previous study has revealed the association between *Enterococci* bone and joint infection and death. Our result first documents this infection as a protective factor. However, certain evidence can explain this relationship. Thompson et al. [26] reviewed the treatment outcomes in 55 patients with enterococcal prosthetic joint infections during a 5-year period. The overall cure rate was about 67%; however, in cases where cure was intended, the overall rate was 80% [26]. Beyond the high cure rate for this type of infection, Fischbacher et al. [27] also documented that the 1-year cumulative mortality was 5.5%, and the 2-year rate was only 7.3%. Thus, our findings confirmed bone and joint infection as a positive prognosis factor for *E. faecium* infection. However, the patients with bone and joint infection in this study had a significantly longer duration of hospitalization than the patients with other infections (median 42 days vs. 12 days, respectively). The enterococcal bone and joint infection thus seems not to impact on mortality but to increase medical costs.

As described above, the present findings indicated that VRE and severe illness were risk factors for mortality. However, it is difficult to select effective agents against *Enterococci* infection, especially in the VRE era, resulting in higher mortality [28]. As per a recent meta-analysis, linezolid treatment or higher-dose daptomycin (≥9 mg/kg) for VRE bacteremia were comparable in terms of the mortality rate. However, linezolid and higher-dose daptomycin were independently associated with lower mortality as compared with lower-dose daptomycin [29]. Therefore, the treatment of *Enterococci* with appropriate antimicrobial regimens could significantly reduce mortality [30].

Besides identifying effective agents against VRE infection, infection control in patients at risk of acquisition of VRE colonization is also important, especially immunocompromised patients, those with hematologic malignancy, organ transplantation, multiple comorbidities, prolonged hospitalization, staying in ICU, and close contact or staying on ward having patients with VRE colonization or infection [31,32]. Alevizakos et al. [33] performed a meta-analysis on the importance of colonization with VRE and found that colonized patients were 24 times more likely to develop a VRE bloodstream infection than non-colonized patients.

The current study has several limitations. First, it is a retrospective study with a small number of patients, which makes it difficult to determine all previous significant factors related mortality and length of stay [13]. Second, our findings were from a university-affiliated hospital, which might be dissimilar from those taken at other types of hospitals. Further studies with a larger and multi-center sample are required to investigate the clinical outcomes of VRE infection.

## 4. Materials and Methods

### 4.1. Study Design

This retrospective study of risk factors related to mortality and clinical outcomes in patients infected with *E. faecium* was performed at the Phramongkutklao Hospital, a teaching hospital with 1200 inpatient beds for Phramongkutklao College of Medicine in Bangkok, Thailand, during the period from 2014 to 2018. The identification of *Enterococci* was performed using conventional techniques. The results of in vitro antimicrobial susceptibility tests for ampicillin and vancomycin with a disk-diffusion method were interpreted based on the Clinical and Laboratory Standards Institute [34]. The institutional review boards at Phramongkutklao College of Medicine and Phramongkutklao Hospital approved the study before its initiation (approval no. Q017b/61_Exp).

### 4.2. Participants

This study included participants (1) aged > 18 years; (2) with results for the first isolate of *E. faecium*; and (3) diagnosed with infection based on the Centers for Disease Control and Prevention/National Healthcare Safety Network (CDC/NHSN) Surveillance Definitions for Specific Types of Infections [35]. Patients who could not be followed up for treatment outcomes, those transferred to another hospital, and those with incomplete medical records were excluded.

### 4.3. Data Collection

The data of the enrolled study subjects were collected from medical records, and the subjects were concealed by coding. The following data were collected: (1) demographic data: gender, age, underlying diseases or comorbidity (malignant tumor, hematologic malignancy, chronic kidney disease, chronic liver function disease, diabetes, neutropenia [defined as neutrophil cell count being <500/mm], connective tissue disease, or cardiovascular disease), duration of admission, ward type when the patients had onset of infection, source of infections (based on CDC/NHSN), receiving anti-*E. faecium* therapy within 72 h from the onset of *E. faecium* infection, or septicemia. (2) Severity of illness: mechanical ventilator use, and Sequential Organ Failure Assessment (SOFA) score, mortality prediction score based on six organ dysfunction systems; SOFA score increased as mortality increased. (3) Mortality rate: in-hospital mortality, 30-day and 90-day mortality. In-hospital mortality was defined as death occurring during the hospital stay; 30- and 90-day mortality was defined as death occurring within 30 and 90 days of a diagnosis of infection.

### 4.4. Statistical Analyses

Descriptive statistics were used for depicting the participants’ characteristics, clinical status, and mortality rate related to *E. faecium* infection. The 1-sample Kolmogorov- Smirnov test was performed for testing the normality of the continuous variables. Chi-square or Fisher’s exact test were performed to analyze the relationship between the categorical variables. Mann Whitney U test (median with interquartile range was used as appropriate) or independent t-test (mean with standard deviation was used as appropriate) were used to compare the median or mean, respectively, of continuous variables. All significant variables in the univariate analysis were considered for the logistic regression analysis based on the backward stepwise (conditional) method. All significant univariate factors were entered first, then considered for elimination based on the probability criteria for stepwise entry and removal.

For the survival analysis, a Cox’s proportional hazard model for 90-day mortality among patients with *E. faecium* infection was employed. Cox’s regression analysis was used for determining the independent risk factors for mortality by selecting the independent variables, that is, those with *p*-values < 0.1 in the univariate analysis, to add into the final Cox’s regression model. The results were reported as hazard ratios with 95% confidence intervals. Data were analyzed with SPSS (IBM Corp., Armonk, NY, USA), and a *p*-value < 0.05 was considered statistically significant.

## 5. Conclusions

The present study indicated the impact of VR-*E. faecium* on mortality and duration of hospitalization. Additionally, severe illness was associated with poor treatment outcomes in patients with *E. faecium* infections. Therefore, in the resistant organism era, using individualized antibiotic regimens with optimal treatment support in severe patients, especially those with VR-*E. faecium* infection, might be a strategy to improve the treatment outcomes.

## Figures and Tables

**Table 1:** Characteristics and clinical outcomes of patients infected with VR-*E. faecium* and VS-*E. faecium*.

Demographic Data	Values		*p-*Value
	VR-*E. faecium* (*n* = 26)	VS-*E. faecium* (*n* = 119)	
Male, *n* (%)	19 (73.1)	61 (51.3)	0.043
Age, median (IQR), y	68.5 (57–80)	74 (59–80)	0.62
Comorbidity, *n* (%)			
Cardiovascular diseases	8 (30.8)	36 (30.3)	0.96
Cerebrovascular diseases	3 (11.5)	23 (19.3)	0.57
End-stage kidney disease	10 (38.5)	14 (11.8)	0.002
Liver disease	6 (23.1)	13 (10.9)	0.11
Solid cancer	7 (26.9)	49 (41.2)	0.18
Hematologic malignancy	3 (11.5)	3 (2.5)	0.07
Neutropenia	4 (15.4)	6 (5)	0.08
Ward, *n* (%)			
Medical-ICU	11 (42.3)	32 (26.9)	0.12
Surgical-ICU	3 (11.5)	14 (11.8)	1.0
Medicine	7 (26.9)	43 (36.1)	0.37
Surgery	5 (19.2)	29 (24.4)	0.58
Others	0 (0)	1 (0.8)	1.0
SOFA score, median (IQR)	7 (7)	5 (7)	0.11
Mechanical ventilator, *n* (%)	16 (61.5)	55 (46.2)	0.16
*E. faecium* bacteremia, *n* (%)	13 (50)	50 (42)	0.46
Polymicrobials, *n* (%)	16 (61.5)	59 (49.6)	0.27
Type of infections, *n* (%)			
Bloodstream infection	7 (26.9)	18 (15.1)	0.159
Cardiovascular system infection	0	2 (1.7)	1.0
Intra-abdominal infection	8 (30.8)	38 (31.9)	1.0
Bone and joint infection	6 (23.1)	5 (4.2)	0.005
Skin and soft tissue infection	2 (7.7)	15 (12.6)	0.539
Urinary tract infection	3 (11.5)	39 (32.8)	0.033
Reproductive tract infection	0 (0)	3 (2.5)	0.633
Clinical outcomes, *n* (%)			
In-hospital mortality	19 (73.1)	59 (49.6)	0.03
30-day mortality	15 (57.7)	46 (38.7)	0.075
90-day mortality	18 (69.2)	56 (47.1)	0.04
Length of hospitalization (day),	69 (38–124)	36 (24–58)	0.001
median (IQR)			

Abbreviations: ICU, intensive care unit; IQR, interquartile range; *n*, number; SOFA, sequential organ failure assessment (SOFA score increased as mortality increased); VR-*E. faecium*, vancomycin-resistant *Enterococcus faecium*; VS-*E. faecium*, vancomycin-susceptible *Enterococcus faecium.*

**Table 2:** Factors predicting 30–day mortality among patients with *Enterococcus faecium* infections by univariate and multivariate analyses.

Variables	Death (61 Cases)	Survivors (84 Cases)	Univariate Analysis		Multivariate Analysis †	
			OR	95% CI	aOR	95% CI
Male, *n* (%)	34 (55.7)	46 (54.8)	1.04	0.54–2.02		
Age ≥ 70 years, *n* (%)	41 (50.6)	40 (49.4)	2.26	1.14–4.47	2.05	0.89–4.71
Comorbidity, *n* (%)						
Cardiovascular diseases	22 (36.1)	22 (26.2)	1.59	0.78–3.27		
Cerebrovascular diseases	10 (16.4)	16 (19)	0.83	0.35–1.99		
End-stage kidney disease	13 (21.3)	11 (13.1)	1.80	0.74–4.34		
Liver disease	12 (19.7)	7 (8.3)	2.69	0.99–7.31		
Solid cancer	21 (34.4)	35 (41.7)	0.74	0.37–1.46		
Hematologic malignancy	3 (4.9)	3 (3.6)	1.40	0.27–7.17		
Neutropenia, *n* (%)	6 (9.8)	4 (4.8)	2.18	0.59–8.09		
SOFA score, *n* (%)						
0–2 points	7 (12.7)	33 (39.8)	-	-	-	-
3–5 points	11 (20)	26 (31.3)	2.00	0.68–5.86	1.52	0.49–4.71
6–9 points	15 (27.3)	13 (15.7)	5.44	1.81–16.39	4.61	1.43–14.87
≥10 points	22 (40)	11 (13.3)	9.43	3.17–28.06	6.94	2.23–21.59
VR-E. faecium, n (%)	15 (24.6)	11 (13.1)	2.16	0.92–5.12	3.64	1.20–11.07
Type of infections, *n* (%)						
Bloodstream infection	16 (26.2)	9 (10.7)	2.96	1.21–7.26		
Intra-abdominal infection	22 (36.1)	24 (28.6)	1.41	0.70–2.85		
Bone and joint infection	1 (1.6)	10 (11.9)	0.12	0.02–0.99	0.09	0.01–0.91
Skin and soft tissue infection	4 (6.6)	13 (15.5)	0.38	0.12–1.24		
Urinary tract infection	17 (27.9)	25 (29.8)	0.91	0.44–1.89		

Abbreviations: aOR, adjusted odds ratio; CI, confidence interval; *n*, number; OR, odds ratio; SOFA, sequential organ failure assessment. SOFA score increased as mortality increased; VR-*E. faecium*, vancomycin-resistant *Enterococcus faecium.* † By backward stepwise (conditional) method.

**Table 3:** Factors predicting 90-day mortality among patients with *Enterococcus faecium* infections by univariate and multivariate analyses.

Variables	Death (74 Cases)	Survivors (71 Cases)	Univariate Analysis		Multivariate Analysis †	
			OR	95% CI	aOR	95% CI
Male, n (%)	40 (54.1)	40 (56.3)	0.91	0.47–1.76		
Age ≥ 70 years, n (%)	52 (70.3)	29 (40.8)	3.42	1.72–6.81	3.56	1.50–8.48
Comorbidity, n (%)						
Cardiovascular diseases	26 (35.1)	18 (25.4)	1.6	0.78–3.27		
Cerebrovascular diseases	14 (18.9)	12 (16.9)	1.15	0.49–2.69		
End-stage kidney disease	16 (21.6)	8 (11.3)	2.2	0.87–5.45		
Liver disease	13 (17.6)	6 (8.5)	2.31	0.83–6.46		
Solid cancer	28 (37.8)	28 (39.4)	0.94	0.48–1.83		
Hematologic malignancy	4 (5.4)	2 (2.8)	1.97	0.35–11.12		
Neutropenia, n (%)	7 (9.5)	3 (4.2)	2.37	0.59–9.55		
SOFA score, n (%)						
0–2 points	9 (13.2)	31 (44.3)	-	-	-	-
3–5 points	16 (23.5)	21 (30)	2.62	0.98–7.04	1.89	0.64–5.59
6–9 points	17 (25)	11 (15.7)	5.32	1.84–15.38	4.40	1.34–14.48
≥10 points	26 (38.2)	7 (10)	12.79	4.19–39.09	9.78	2.93–32.70
VR-E. faecium, n (%)	18 (24.3)	8 (11.3)	2.53	1.02–6.27	7.35	1.79–30.21
Type of infections, n (%)						
Bloodstream infection	19 (25.7)	6 (8.5)	3.74	1.40–10.3		
Intra-abdominal infection	25 (33.8)	21 (29.6)	1.22	0.60–2.45		
Bone and joint infection	1 (1.4)	10 (14.1)	0.08	0.01–0.67	0.034	0.02–0.49
Skin and soft tissue infection	6 (8.1)	11 (15.5)	0.48	0.17–1.38		
Urinary tract infection	21 (28.4)	21 (29.6)	0.94	0.46–1.93		

Abbreviations: aOR, adjusted odds ratio; CI, confidence interval; *n*, number; OR, odds ratio; SOFA, sequential organ failure assessment. SOFA score increased as mortality increased; VR-*E. faecium*, vancomycin resistant *Enterococcus faecium.* † By backward stepwise (conditional) method.

**Table 4:** Multivariate Cox proportional hazard regression analysis of 90-day survival among patients with *E. faecium* infections (*n* = 145).

Variable	Hazard Ratio	95% CI	*p*-Value
Age ≥ 70 years	1.61	0.92–2.82	0.093
VR-*E. faecium* infection	1.91	1.09–3.37	0.024
SOFA score			
0–2	Reference	Reference	-
3–5	1.06	0.47–2.52	0.894
6–9	2.69	1.15–6.29	0.022
≥10	3.71	1.70–8.13	0.001
Bloodstream infection	1.02	0.57–1.83	0.949
Bone and joint infection	0.08	0.01–0.62	0.015
